# Oncolactation for Patients with Breast Cancer: Executive Summary from the American Society of Breast Surgeons

**DOI:** 10.1245/s10434-025-17742-7

**Published:** 2025-07-08

**Authors:** Helen M. Johnson, Megan E. Miller, Katrina B. Mitchell

**Affiliations:** 1 Department of Breast Surgical Oncology, The University of Texas MD Anderson Cancer Center, Houston, TX USA; 2 Department of Surgery, University Hospitals Cleveland Medical Center, Case Western Reserve University School of Medicine, Cleveland, OH USA; 3 Ridley Tree Cancer Center, Sutter Health, Santa Barbara, CA USA

**Keywords:** Breast cancer, Oncolactation, Breastfeeding, Lactation, Pregnancy, Postpartum, Survivorship, Screening

## Abstract

Despite increasing rates of pregnancy-related and postpartum breast cancers and growing recognition of oncofertility as an important component of survivorship, few guidelines exist to guide surgical oncologists in managing lactation during or after breast cancer treatment. To address this need, the American Society of Breast Surgeons developed evidence-based oncolactation recommendations for breast cancer patients. This review outlines the management of patients with breast cancer diagnosed during pregnancy or lactation, and provides guidance regarding surgery, radiation, and systemic therapy. It also reviews strategies for supporting lactation following breast cancer treatment and discusses screening in the pregnant and lactating population.

Oncolactation is defined as the intersection of lactation and oncology care in women diagnosed with cancer during pregnancy; postpartum mothers diagnosed with cancer while breastfeeding; and survivors who wish to breastfeed after treatment. At present, limited recommendations exist for managing oncolactation.

Pregnancy-related breast cancer (PrBC) and postpartum breast cancer (PPBC) rates have increased, partly due to women delaying childbearing.^1,2^ In addition, young women with breast cancer may desire childbearing after treatment. Healthcare providers should understand and communicate the impact of each breast cancer treatment modality on future lactation.^3^

A recent survey of breast surgeons demonstrated strong American Society of Breast Surgeons (ASBrS) member interest in expanding formal education for lactation and developing management guidelines.^4^ To address this clinical need, the ASBrS developed evidence-based oncolactation recommendations for breast cancer patients. This review aims to describe guidelines for breast cancer screening and diagnosis during pregnancy and lactation and to outline the management of patients with breast cancer diagnosed during pregnancy or lactation, as well as those with a history of breast cancer treatment who desire to breastfeed.

## Methods

In 2021–2022, the ASBrS conducted a survey of its members to characterize existing sources of information on lactation; assess the need for additional knowledge; and understand member preferences for expanded education.^4^ The results of this survey highlighted a strong interest in more formal education, particularly through the development of evidence-based management guidelines.

In February 2023, the ASBrS Research Committee proposed the creation of a statement on oncolactation for breast cancer patients. The Committee invited two ASBrS members with expertise in oncolactation (HMJ and KBM) to lead the initiative. In accordance with Committee policy, a literature review of all articles relating to the intersection of lactation, breast surgery, and breast cancer was conducted, inclusive of large national and international datasets, clinical trials, basic science publications, and recent guidelines from professional societies in pertinent fields. An outline was drafted and submitted for review by the Committee. After incorporation of suggested revisions, the outline was reviewed by the ASBrS Board of Directors and approved. A full statement was then drafted by the lead authors, formatted per ASBrS requirements, and reviewed by the organization’s medical editor. After approval by the committee, the document was submitted to the board. After incorporating the board’s suggested edits, the revised document was approved and posted on the ASBrS website for member comment. Minor revisions were made based on such comments and the final version was resubmitted to the board. The board approved the resource guide, and the document was posted on the ASBrS website.^5^

## Recommendations for Oncolactation Management in Breast Cancer Patients

### Breast Cancer Screening and Diagnosis during Pregnancy and Lactation

Lactational status affects breast cancer screening and diagnosis.^6^ Pregnancy and lactation induce physiological changes in the breast, altering its imaging appearance. Therefore, examinations must be interpreted contextually, and lactating women may require supplemental imaging modalities. While statements by the American College of Radiology (ACR)^7^ and the American College of Obstetricians and Gynecologists (ACOG)^8^ describe the safety of breast imaging during pregnancy and lactation, delays in breast cancer diagnosis and treatment may occur due to avoidance of imaging during these periods and lack of clinical suspicion for malignancy. It is crucial that women not forgo or delay indicated imaging owing to pregnancy or lactational status, particularly given the aggressive nature of PPBC.^1^ Key recommendations are summarized in Table 1.

**Table 1:** Key recommendations for breast cancer screening and diagnostic evaluation in pregnant or lactating patients

Breast cancer screening should not be delayed or forgone during pregnancy and lactation if indicated based on the patient’s age or risk profile.
During pregnancy, mammography without fetal shielding and breast ultrasonography are safe; however, breast magnetic resonance imaging (MRI) with gadolinium contrast should be limited owing to sparse fetal safety data.
All breast imaging modalities, including MRI, are safe during lactation and do not require interruption of breastfeeding.
Patients should breastfeed or express breastmilk (e.g., pump) immediately prior to imaging studies to optimize sensitivity.
Percutaneous core needle biopsy is safe in pregnancy and lactation. Patients should resume breastfeeding normally after the procedure.

#### Imaging Modalities

Mammography and ultrasonography are safe during pregnancy and lactation. Contrary to past recommendations, the ACR now advises against the use of fetal shielding, because it causes scatter and increases fetal radiation dose; without a shield, the exposure is otherwise negligible.^9,10^ There are no data on contrast-enhanced mammography in these patient populations. Because iodinated contrast is known to cross the placenta but fetal safety is unknown, ACOG recommends avoiding contrast in pregnant patients unless its use would significantly impact management.^8^

Magnetic resonance imaging (MRI) with gadolinium is not recommended by the ACR during pregnancy, because gadolinium crosses the placenta and may be teratogenic.^7^ Given the paucity of fetal safety data, the ACOG acknowledges that the use of gadolinium in pregnancy is controversial and advises that its use be reserved for patients for whom “the benefits clearly outweigh the possible risks.”^8^ Lactation may reduce the sensitivity of breast MRI, but it is a safe and effective screening and diagnostic modality in the postpartum population.^9^

To optimize sensitivity, patients should breastfeed or express breastmilk (e.g., pump) immediately prior to breast imaging. They can breastfeed normally after any study, including MRI with gadolinium contrast and mammography with intravenous iodinated contrast.^10^

#### Screening

Although there are limited data on screening pregnant and lactating women, expert guidelines recommend that women undergo screening mammography during pregnancy and lactation as indicated based on age and risk profile. Pregnant and lactating high-risk women of any age and those older than 40 years may benefit from supplemental screening breast ultrasound.^7^ For patients in the process of weaning, it is reasonable to defer screening for 1 to 2 months.

High-risk women for whom screening breast MRI is indicated should forgo breast MRI during pregnancy and resume MRI after delivery. High-risk women who plan to breastfeed for longer than 6 months should not alter their screening MRI schedule; for high-risk women in the process of weaning, MRI should be postponed until 6 to 8 weeks after weaning.^7,11^

#### Diagnostic Evaluation

Ultrasonography is the first-line diagnostic imaging study for pregnant and breastfeeding women with a palpable mass or other concerning symptoms; mammography can provide additional information as indicated.^7^

Percutaneous core needle biopsy with local anesthetic and titanium clip placement is safe in pregnancy and lactation. No interruption of lactation is needed. There is a minimally increased risk of bleeding owing to the vascularity of the lactating breast.^12^ The risk of milk fistula is very low and should not preclude necessary biopsies.^13^ This risk may be decreased by positioning the biopsy tract as far from the nipple areolar complex as possible. It is normal for biopsy sites to leak milk for several days before self-resolving; they should not be closed with suture or surgical adhesive owing to risks of fluid collection and mastitis.

### Breast Cancer Management during Lactation

#### Staging Studies

Staging studies such as computed tomography (CT) with iodinated contrast and bone scan do not require interruption of breastfeeding. Fluorodeoxyglucose (FDG)-positron emission tomography with CT (PET-CT) requires 12 hours of separation of mother and infant due to external radioactivity, but FDG is not excreted in breastmilk. The mother should express milk during this time and another person can feed it to the infant.^14,15^

#### Treatment

It is safe to breastfeed from the affected breast until treatments begin, although subsequent therapies will impact lactation. Lactating patients diagnosed with PPBC require attention for maintaining or ceasing breastmilk production based on individual clinical considerations. If surgery or radiation is anticipated early postpartum, consider augmenting milk production in the unaffected breast and reducing production in the affected breast.^3^ Autocrine and paracrine mechanisms regulate milk synthesis in each breast individually, so continued feeding from one breast does not promote persistent milk production in the other.^16^ Key recommendations are summarized in Tables 2 and 3.

**Table 2:** Key recommendations for breast cancer surgery in pregnant or lactating patients

Do not delay nonelective breast surgery in the postpartum period for weaning. Milk fistulae are rare when lactation is managed appropriately, and complication rates are no higher than average.
While data are limited, patients should express and discard milk for 24 hr following receipt of blue dye (i.e., methylene blue, isosulfan blue) and/or radiotracer for sentinel lymph node surgery. No breastfeeding interruption is needed after use of indocyanine green. There are no lactational safety data for superparamagnetic iron oxide.
Because of the potential teratogenicity of methylene blue and anaphylactic risk associated with isosulfan blue, single tracer lymphatic mapping with radioactive colloid is preferred during pregnancy. Limited data support the safety of indocyanine green during pregnancy, but similar data do not exist for the use of superparamagnetic iron oxide.
Follow evidence-based recommendations for the perioperative management of lactating women, including avoidance of unsafe medications and minimization of breastfeeding interruptions.

**Table 3:** Key recommendations for radiation therapy and systemic therapy in pregnant or lactating patients

Women should not breastfeed or express milk (e.g., pump) from the affected breast while undergoing radiotherapy; however, they may safely feed from the unaffected breast.
Cytotoxic chemotherapy and endocrine therapy transfer into breastmilk and make it unsafe for infant consumption.
There are currently no data on the safety of anti-HER2 agents, immunotherapy, and CDK4/6 inhibitors during lactation.

#### Surgery

Do not delay surgery for cancer due to concern for complications, such as milk fistula, which is rare when patients continue to breastfeed normally after invasive procedures.^13,17^ The lactating breast has excellent blood flow and contains antimicrobial factors that may protect against infection.^18^ Surgery should also not be delayed for weaning, as postlactation involution is a complex process that can take months to years.^19,20^

##### Breast-Conserving Surgery

- There are minimal data on the use of wire and nonwire localization methods during lactation. Breastfeeding or pumping with a wire in place may risk displacement. However, radioactive I-125 seeds, magnetic seeds, radar reflectors, and other localizing devices are embedded in tissue and are not known to be at increased risk of migration during lactation. To prevent exposure of the infant to radioactivity, breastfeeding should be avoided after insertion of a radioactive seed and can resume after its removal.^21^
- To prevent fluid collection in the setting of high milk production, consider leaving a short-term drain to *gravity*. Drains should not be left to suction, because this will promote excessive stimulation of milk removal and prolong the need for a drain.^16^ If a patient is further postpartum and/or has baseline low production, a drain may not be necessary.
- For patients desiring continued or future breastfeeding, minimize the extent of surgery and parenchymal disruption (e.g., large-volume local tissue rearrangement or oncoplastic reconstruction) and place the incision far from the nipple areolar complex if possible.^22^

##### Mastectomy

- This procedure removes 95% or more of functional breast tissue; therefore, postoperative drains can be placed to suction.
- Skin and/or nipple-sparing mastectomy with reconstruction can be performed safely.

##### Axillary Staging

- Radiotracer (Tc 99 sulfur colloid) lactational safety information is limited. Expert guidelines and prescribing information advise that patients express and discard milk for 24 hr after sentinel lymph node surgery with radiotracer.^23,24^ This may be a conservative estimate, given that no interruption in breastfeeding is recommended after intravenous injection.^25^ While Tc 99 crosses the placenta, modeled fetal radiation exposure from lymphatic mapping (1.4 µGy-4.3 mGy) is well below the threshold of concern for fetal harm (100–200 mGy);^26,27^ available data in pregnant women have not identified specific drug-related risks of major birth defects, miscarriage, or adverse maternal or fetal outcomes.^24^ Same-day administration of radiotracer is recommended to facilitate use of the lowest possible dose and minimize maternal, neonatal, and infant exposure.^26–28^
- There are no data on the relative infant dose of methylene blue or isosulfan blue after intradermal or intraparenchymal injection in a lactating breast. Infant ingestion of methylene blue has been associated with hemolytic anemia; no safety data are available for isosulfan blue in children.^29–32^ InfantRisk advises that patients express and discard milk for 24 hr after receipt of intravenous methylene blue.^33^ Isosulfan blue is generally avoided during pregnancy due to the 2% risk of life-threatening anaphylactic reactions.^29^ When used intra-amniotically, methylene blue can cause serious adverse prenatal and neonatal complications; however it is unknown whether this translates to fetal risk when administered at the significantly lower dose and via the subareolar route used for lymphatic mapping.^26–28^ While small series have reported safe and feasible administration of both blue dyes during pregnancy, single tracer mapping with radioactive colloid is preferred to minimize maternal and fetal risks, and is supported by several international societies’ guidelines.^26,27,34^
- Indocyanine green (ICG) is safe in lactation and no breastfeeding interruption is required.^35^ Indocyanine green does not appear to cross the placenta and is safe after intravenous administration, though there are no specific data on lymphatic mapping during pregnancy.^36,37^
- The safety of superparamagnetic iron oxide during pregnancy and lactation is unknown.^38^

##### Perioperative Considerations

- Avoid narcotics, as all opioids transfer to breastmilk. If necessary, morphine and hydromorphone are preferred owing to poor oral bioavailability. Codeine, tramadol, and meperidine are not recommended in lactation.^39^
- Use intravenous fluids judiciously to minimize breast edema, which can interfere with latching and effective pumping.^40^
- Advise patients to pump the unaffected breast prior to surgery to maintain milk production. However, patients should not pump the affected breast as milk removal stimulates increased milk production.^16^
- Minimize separation of mother and child.^41^
- Breastfeeding is safe after anesthesia when the patient is alert enough to hold and latch her baby or utilize a breast pump.^39^
- Local anesthetics are safe and do not require interruption of breastfeeding.^39,42^

#### Radiation Therapy

Patients should not breastfeed from or pump the affected breast while undergoing partial or whole breast radiation due to risks of wounds and abscess, interference with radiation volumetric plans, and apoptosis of functional cells.^43^ Patients can breastfeed without interruption from the unaffected breast while undergoing radiation therapy.

#### Systemic Therapy

- Endocrine therapy is contraindicated during lactation as agents readily transfer to breastmilk and may inhibit milk production and impact estrogen metabolism in infants.^3,21,44–48^
- Cytotoxic chemotherapy agents are excreted in breastmilk, which is not safe for infant consumption and should be discarded. Cabergoline, an indirect prolactin antagonist, can be used off-label to halt milk production when chemotherapy begins. Most commonly, 0.25–0.5 mg by mouth every 72 hours is used; most patients require one to three doses for reduction of engorgement and milk flow.

Women who desire to express breastmilk to maintain production should be advised about the high rate of complications, including mastitis, nipple injury, decreased milk volume, change in breastmilk microbiome, and infant disinterest in return to nursing at the breast after completion of chemotherapy.^22^ If women still desire to pump milk, they should follow recommendations from an oncologic pharmacist regarding the safety of feeding any milk to their child.^49,50^

- Targeted anti-HER2 agents and immunotherapies, such as pembrolizumab, likely are minimally excreted in breastmilk owing to their large molecular weight; however, no safety data exist and breastfeeding is not recommended for women receiving these drugs.^51–53^
- Breastfeeding is not recommended for women taking cyclin-dependent kinase 4 and 6 (CDK 4/6) inhibitors owing to the lack of safety data.^54–56^

### Lactation Following Breast Cancer Treatment

Breastfeeding does not increase breast cancer recurrence risk.^57^ In fact, breastfeeding reduces the risk of a primary breast cancer, and so could reduce recurrence risk in women diagnosed with breast cancer^58^; more studies are needed.

Treatment for breast cancer will impact future lactation, but breastfeeding is possible for many breast cancer survivors.^59^

#### Surgery

The affected breast will not be functional after a mastectomy, regardless of technique (including nipple-sparing). A supplemental nursing system (feeding tube at the breast) or nonnutritive suckling after nipple-sparing mastectomy should be avoided owing to risk of nipple and/or skin trauma and subsequent wound formation. If significant engorgement or milk production is noted after mastectomy, the patient should be referred to a surgeon for discussion regarding the volume of residual breast parenchyma. Partial mastectomy has limited impact on breastfeeding unless central retroareolar ducts are excised.

#### Radiation Therapy

Radiation therapy causes apoptosis and fibrosis in the breast parenchyma, precluding normal mammogenesis and lactogenesis during pregnancy. Therefore, the affected breast will be unable to synthesize milk normally. Some breasts are completely unable to produce milk, whereas others may produce a limited quantity. Counsel patients against feeding or expressing milk from a previously irradiated breast because of the risk of skin maceration and wound formation, as well as changes in breastmilk composition.^3,43,60^ Similarly, counsel patients against using a supplemental nursing system on a previously irradiated breast.

#### Systemic Therapy

Systemic chemotherapy may alter the functional capacity of residual breast tissue.^61^ The POSITIVE trial provides short-term evidence supporting the oncologic safety of interrupting endocrine therapy for pregnancy and/or breastfeeding.^62^

##### Risk Reducing Mastectomy and Contralateral Prophylactic Mastectomy

Women of childbearing age considering contralateral prophylactic mastectomy (CPM) or risk reducing mastectomy (RRM) should receive comprehensive counseling, including discussion about oncolactation. Shared decision-making principles should guide the discussion regarding RRM and CPM, focusing on the patient’s individualized risk of contralateral breast cancer, respectively. Surgeons should educate patients that CPM does not improve survival.^63,64^ Women should be counseled that not breastfeeding increases maternal and infant morbidity and mortality in acute and chronic health conditions.^65^ In addition, breastfeeding significantly reduces ovarian cancer risk in BRCA carriers.^66^ Therefore, patients can consider postponing CPM until after childbearing and continue appropriate cancer screening in the interim. Indeed, the risk of metastatic recurrence after PPBC is greater than that of a new contralateral breast cancer in the short duration of a woman’s childbearing years.^67,68^ Key recommendations are summarized in Table 4.

**Table 4:** Key recommendations for oncolactation counseling for women of childbearing age considering contralateral prophylactic mastectomy or risk reducing mastectomy

Women of childbearing age considering contralateral prophylactic mastectomy (CPM) or risk reducing mastectomy (RRM) should receive comprehensive counseling, including discussion about oncolactation.
Shared decision-making principles should guide decisions about CPM and RRM, focusing on the patient’s risk of primary or contralateral breast cancer, lack of survival benefit, and risks of not breastfeeding.
Women can consider delaying RRM/CPM until after completion of childbearing, with high-risk screening pursued in the interim.

##### Lactation Support and Resources

Key recommendations are summarized in Table 5. Physicians trained in breastfeeding medicine, a specialty dedicated to all aspects of lactation care, can play a significant role in the multidisciplinary breast oncology team and offer patients prenatal and postpartum support. They can be located through the Academy of Breastfeeding Medicine or the Institute for the Advancement of Breastfeeding and Lactation.^69,70^ If a breastfeeding medicine physician is not available, a non-physician provider with lactation training, such as an International Board-Certified Lactation Consultant, may be consulted.^71^

**Table 5:** Key recommendations for lactation support for breast cancer patients

Breast cancer survivors, patients with pregnancy-related breast cancer (PrBC), and patients with postpartum breast cancer (PPBC) who are breastfeeding at diagnosis should receive prompt referrals to a breastfeeding medicine physician or board-certified lactation consultant. Oncolactation is a critical component of multidisciplinary care and survivorship for these patients.
Breastfeeding is possible from the contralateral breast for many breast cancer survivors. It should be avoided on the affected breast after breast conserving therapy and is not possible after a mastectomy. Prior chemotherapy may decrease milk production. Patients who are unable to produce sufficient milk for healthy infant growth should be counseled about utilizing donor breastmilk.

Strategies to increase breastmilk production include hand expression, pumping, frequent feedings, and galactogogues; the latter should be discussed with breastfeeding providers and oncology survivorship owing to potential interactions with endocrine therapy and/or phytoestrogen mechanism of action.^22,72^ Patients unable to produce adequate volumes of breastmilk or those who require weaning for treatment may benefit from donor milk through milk banks or informal milk sharing.^73,74^

As systemic therapy agents evolve, safety profiles for agents during lactation are available at the National Institute of Health’s LactMed database^75^ or by consulting the InfantRisk Center,^76^ which also offers an app for healthcare providers (InfantRisk HCP).
